# A Risk-Based Approach in Rehabilitation of Water Distribution Networks

**DOI:** 10.3390/ijerph19031594

**Published:** 2022-01-30

**Authors:** Gema Sakti Raspati, Stian Bruaset, Camillo Bosco, Lars Mushom, Birgitte Johannessen, Rita Ugarelli

**Affiliations:** 1 Group Water and Environment, SINTEF Community, S.P. Andersens vei 3, N-7034 Trondheim, Norway; stian.bruaset@sintef.no (S.B.); camillo.bosco@sintef.no (C.B.); rita.ugarelli@sintef.no (R.U.); 2 Group Mathematics and Cybernetics, SINTEF Digital, Forskningsveien 1, N-0373 Oslo, Norway; lars.mushom@gmail.com; 3 Trondheim Municipality, Erling Skakkes Gate 14, N-7013 Trondheim, Norway; birgitte.johannessen@trondheim.kommune.no; 4 Department of Civil and Environmental Engineering, Norwegian University of Science and Technology (NTNU), S.P. Andersens vei 5, N-7034 Trondheim, Norway

**Keywords:** risk-based rehabilitation, risk analysis, pipe break, Random Forest, reliability

## Abstract

A risk-based approach to support water utilities in terms of defining pipe rehabilitation priorities is presented. In a risk analysis in the risk management process, the probability that a given event will happen and the consequences if it does happen have to be estimated and combined. In the quantitative risk analysis, numerical values are assigned to both consequence and probability. In this study, the risk event addressed was the inability to supply water due to pipe breaks. Therefore, on the probability side, the probability of pipes breaking was assessed, and on the consequence side, the reduced ability to satisfy the water demand (hydraulic reliability) due to pipe breakage was computed. Random Forest analysis was implemented for the probability side, while the Asset Vulnerability Analysis Toolkit was used to analyse the network’s hydraulic reliability. Pipes could then be ranked based on the corresponding risk magnitude, thereby feeding a risk evaluation step; at this step, decisions are made concerning which risks need treatment, and also concerning the treatment priorities, i.e., rehabilitation priorities. The water distribution network of Trondheim, Norway, was used as a case study area, and this study illustrates how the developed method aids the development of a risk-based rehabilitation plan.

## 1. Introduction

The water supply is considered to be one of the critical infrastructure sectors whose assets, systems and networks play significant roles in modern society [1]. Incapacitation or impairment of the water supply system will impose catastrophic effects on public health, economy, security, or any combination thereof. The water supply system must provide water to the customers in good quality, quantity, and continuity. The water distribution network (WDN) is crucial for ensuring a well-functioning centralized water supply system. Aging of the WDN has become one of the major issues that demand attention to uphold the objectives of drinking water provision [2]. This issue requires a long-term rehabilitation strategy in which plans for maintaining or upgrading the WDN are systematically set. Water utility providers are often challenged to set their priorities correctly, e.g., due to budget and resource limitations. The implementation of infrastructure asset management (IAM) principles may help the water utility providers make better decisions under such constraints, avoid reactive approaches, and improve the process of WDN rehabilitation planning.

IAM applied to urban water systems consists of a multidisciplinary approach to guide a water utility in providing the set level of service in an efficient, effective, and economic way. In IAM, three decision levels are identified in an organization: a strategic level, driven by corporate and long-term views; a tactical level, where the intermediate managers in charge of the infrastructures need to select what the best medium-term intervention solutions are; and an operational level, where short-term actions are planned and implemented [3]. At the tactical level, rehabilitation decisions are taken and involve some aspect of performance/cost/risk trade-off. Risk management is the process of identifying, quantifying, and managing the risks that an organization faces. ISO 31000 is a standard for risk management. In ISO 31000, the focus is on best practice principles for implementing, maintaining, and improving a framework for risk management. According to ISO 31000, a risk management process starts with the establishment of a team, and it covers the following steps: (i) establishing the context; (ii) risk assessment; (iii) risk treatment; and (iv) monitoring and review. The risk assessment step involves risk identification to identify risk events preventing an organization from achieving a set goal; risk analysis aims to understand the sources and causes of the identified risks by studying probabilities and consequences to assess the level of risk and conducting a risk evaluation to compare risk analysis results with risk criteria to determine if the computed risk is tolerable.

The result of the risk assessment step consists of a categorization of the risk events that are tolerable and those for which immediate actions must be taken. At a tactical level, the water operator must assess what risk is tolerable by balancing the risk with the system performances and the available resources to treat the risk, and thereby define the priorities of intervention. Risk assessment is a process that in many cases is not (at least not adequately) performed, even if risk management is implemented by water utility providers. One of the main objectives of this paper is to facilitate the use of risk assessment by providing a practical example of its applications in a real case study.

The assessment approach adopted for quantifying risk needs to be selected with respect to the specific scope of the risk analysis, i.e., with consideration of qualitative, semi-quantitative, or quantitative measures of risk and determination of whether the risk analysis comprises the complete water utility system or some subsystem(s) of it. Risk is traditionally expressed by the combination of the severity of the consequences induced by unwanted events (C) and the likelihood (i.e., probability, P, or frequency, f) of the event to happen. In this study, the risk event addressed is the “inability to supply water due to pipe break”. Therefore, on the probability side, the probability of pipes breaking (structural reliability) is assessed, and on the consequence side, the number of nodes disconnected and the corresponding unsupplied flow owing to a pipe break event are computed.

The success of the rehabilitation strategy is greatly dependent on the accuracy of the pipe failure forecasting model in use. Thus, a number of physically and statistically based water main prediction models have been developed in the last 40 years. The review of Kleiner and Rajani [4] presents an overview of the statistical models developed prior to 2001. Since the review of Kleiner and Rajani [4], the knowledge about machine learning techniques has become popularized in the water sector and has been adopted in pipe failure forecasting modelling. These modelling techniques include, but are not limited to, genetic algorithms [5], artificial neural networks [6], Random Forest analysis [7], boosted decision trees [8], fuzzy logic, support vector machines, etc. Machine learning and statistical methods have become an invaluable tool for forecasting [9] and lifetime analysis [10]. The applications include financial markets [11], modelling of dynamical systems [12], and predictive maintenance [13]. Predicting the remaining service life of a physical component provides a useful decision support regarding whether to rehabilitate or to replace the component. This has an obvious economic benefit while also ensuring the safety and productivity of the system. Powered by increased data collection and the integration of physical and digital systems in industrial applications, data-driven methods are valuable, for instance, in production facilities, electricity grids, and offshore activities [13]. Recent trends show that the use of data-driven models is becoming more common for water resource management [14].

Traditionally, physical models are used to capture the dynamics of a system in the form of differential equations. However, as in the case of breakage of water pipes [15], the physical models are not able to capture the underlying physics. This may be due to complex interactions and unmodeled effects. In the case of highly accurate calibration of the hydraulic models, physical-based simulation analysis can be adopted to identify pipelines that require rehabilitation [16]. The advantage of physical models is that they are highly interpretable and they do not require a direct observation of a system, i.e., they can be extrapolated to unseen areas of the data domain. On the other hand, machine learning models provide a flexible framework that can adapt to the data and often yield excellent predictive performance. Such models require little prior knowledge about the system, but they can be harder to interpret. Deep learning models [17] have, in recent years, achieved unprecedented results, but they act as a “black-box” for the practitioner. This makes it difficult to adopt such methods in industrial applications where the predictions leading to decisions must be held accountable. Efforts have been made in combining the physical and data-driven models [18].

An alternative to cope with the lack of transparency of flexible data-driven models would be to use a simple model that is inherently interpretable. For instance, this could be a linear model or a decision tree. However, the interpretability comes at the cost of worse predictive performance. Random Forest (RF) analysis can be used as a tradeoff between interpretability and flexibility [17]. RF is an ensemble method that deploys a multitude of decisions trees in training and aggregates their predictions [19]. RF has become a popular model that achieves reasonable predictions with very little requirements for configuration and has also been previously used to model water distribution networks (WDN) [8].

Among the many elements in a WDN, pipes are the primary components for conveying water to customers. Each of these pipes can suffer failure (e.g., intentional due to maintenance or unintentional due to breakage) that decreases network functionality depending on the importance of the pipe, as well as impacting the provision of water supply for the customers [20]. The criticality of a pipe is usually assessed by quantifying the decrease in the network functionality in a WDN reliability analysis. The reliability concept has been a central concept in WDN design, operation, and maintenance, and was developed as a continuation of the classical reliability concept that divides reliability into mechanical and hydraulic reliability [21,22] and uses various indices and methods of assessment. In general, the mechanical reliability puts emphasis on the network topology by evaluating system connectivity under given failure conditions. Pipe failure statistics [23] and probability [24] are later incorporated into the mechanical reliability analysis to better indicate the criticality of pipes in a WDN, and some studies include the water availability aspect in their simulation of pipe failure and repair events [25]. On the other hand, hydraulic reliability refers to the ability of a system to meet the requirements of water flow and pressure. Quantification of the hydraulic reliability involves results from hydraulic simulation through the use of nodal pressure [26] or even more complex approaches, e.g., unsupplied demand, economic loss, pressure deficiency, water quality [27], and energy [28].

The objective of this study is to develop a risk-based approach for prioritizing pipe rehabilitation. The paper discusses, in detail, the steps involved in the approach, which include: (1) identification of risk event for the risk analysis, i.e., inability to supply water due to pipe breaks; (2) assessment of the probability of a pipe break (P) by means of a machine learning method (RF); (3) Consequence (C) assessment conducted with the use of the Asset Vulnerability Analysis Toolkit (AVAT) that evaluates the topological importance of each pipe in a water distribution network and estimates the hydraulic reliability of each pipe with the support of complex network theory; (4) risk evaluation at pipe level using a risk matrix approach. A risk matrix is a method that provides an approximation to a quantitative relation between Consequence (C) and Probability (P). The risk matrix enables:

- Estimation of a risk level of identified risk events;
- Setting of the risk criteria: the levels of acceptable risk;
- Discrimination between three levels of risk associated with acceptance criteria:
  ○ Low (acceptable);
  ○ Medium (tolerable);
  ○ High (not acceptable).

Subsequently, (5) ranking of risk events takes place according to their severity/level in the risk matrix, thereby enabling prioritization for the pipe rehabilitation plan.

The paper is structured in sections. First, the risk assessment methodology is described, including the information on the case study, and how the probability and consequences are calculated. Secondly, the results of probability, consequence, and risk assessment, as well as the pipe ranking, are presented. Finally, the results are discussed and compared with other studies, including limitations and potential improvements of the proposed method, followed by the main conclusions and future perspectives of the study.

## 2. Materials and Methods

### 2.1. Description of the Case Study

**Figure 1:**
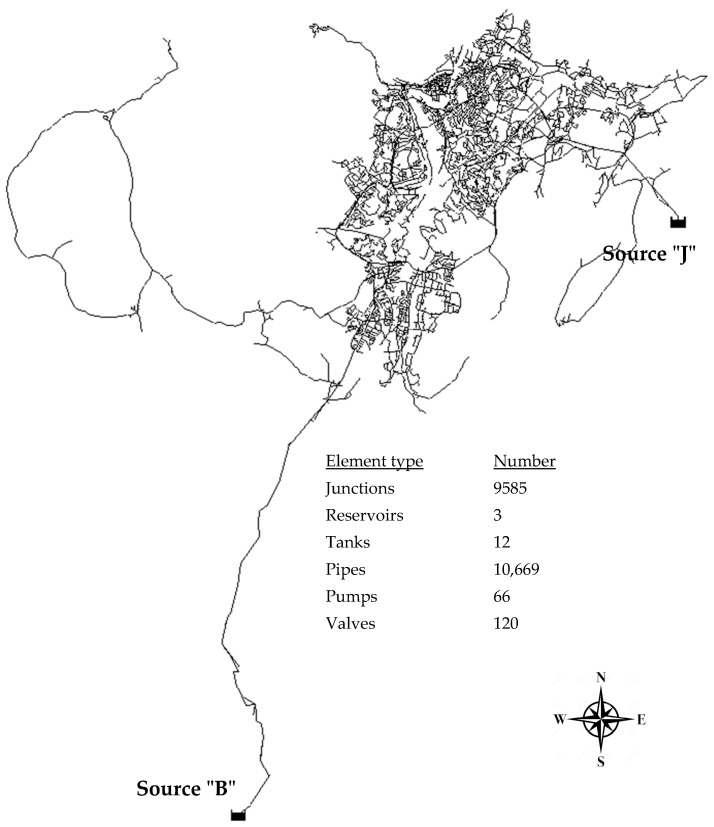
Water distribution network of Trondheim used as the case study.

The city of Trondheim, in Trøndelag County, Norway, is the third most populous municipality in Norway with ~220,000 inhabitants. The water distribution system of the city was used as the case study for the proposed risk-based rehabilitation method in this study. Figure 1 shows the water distribution network (WDN) of Trondheim and general information of the system topology.

There are two main sources of the drinking water: sources “J” and “B”. The daily operation of the WDN involves the simultaneous operation of both sources, but the WDN must also allow uninterrupted service should one of the two sources fail. This imposes a complex operational strategy and operational flexibility (e.g., a control system for the pumps, valves, and tanks). Of particular interest in this study, a risk-based rehabilitation approach shall be proposed to help the municipality decide their pipe rehabilitation plan based on a set of risk criteria presented in the following sections.

### 2.2. Risk Assessment Methodology Applied in This Study

**Figure 2:**
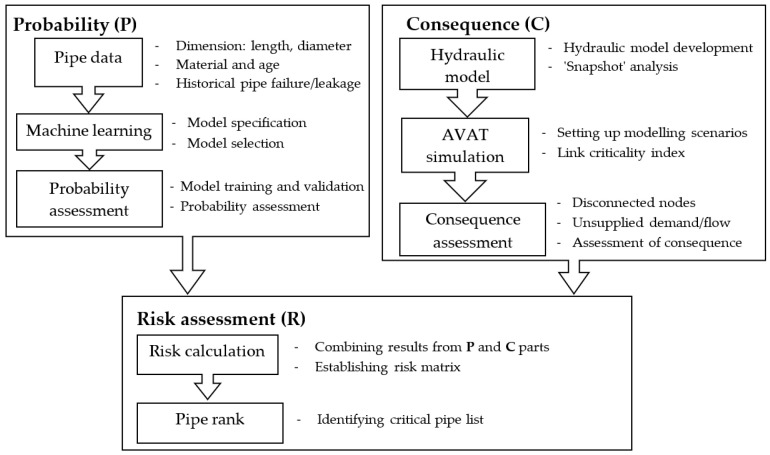
Workflow of Probability (P), Consequence (C), and Risk Assessment (R) applied in the study.

Figure 2 depicts the methodology of the risk-based rehabilitation method proposed in this study. The method encompasses two independent components, i.e., Probability (P) and Consequence (C) assessment, that contribute to the Risk Assessment (R). The probability assessment involves a machine learning method that assesses the pipe failure probability based on the historical data. The consequence assessment requires a hydraulic model to evaluate the criticality of each pipe for the operation of the WDN and to quantify the flow conveyed by each pipe. The following sections provide in-depth explanation of the approach implemented in the risk-based pipe rehabilitation method.

### 2.3. Probability Assessment (P): Machine Learning Model

To model the probability of a pipe breakage, a machine learning classifier for a binary problem was employed. Instead of predicting whether a pipe will break or not, the classifier will return the estimated probability of a pipe breaking. Random Forest (RF) was chosen to assess the response probability of a pipe breaking within a five-year horizon. The question of imbalanced training data was addressed when selecting the best model as pipe break is a rare event.

#### 2.3.1. Model Specification

Decision trees are a class of supervised models applicable for both regression and classification problems [29]. They work by splitting the feature space into a set of rectangles, and then making a prediction for each one. Tree-based models are conceptually simple yet powerful. A decision tree is usually built using a greedy strategy called recursive binary splitting. Then, at each node, a feature and a threshold are selected to result in two branches. The feature and threshold are chosen to minimize a loss function, such as the *mean squared error* for regression and the *Gini index* for classification. This process is repeated until a stopping criterion, e.g., a minimum limit of datapoints in the terminal nodes, is met. A cost–complexity pruning strategy is usually used to reduce the size of the tree.

An advantage of decision trees lies in their simplicity, which makes them computationally efficient during training and easily interpretable during decision-making. In fact, one could visualize the model itself and directly infer which relationships in the data are responsible for a prediction. Unfortunately, trees typically do not have the same predictive accuracy as more flexible models. Moreover, they can be non-robust and have high variance in the predictions. To address these shortcomings, bootstrap aggregation, also called *bagging*, can be applied [30]. Bootstrap is a general statistical technique usually applied to estimate the variance of a quantity of interest. It approximates the distribution of the data by sampling the observed data with replacement data. In bagging, bootstrap sampling is used to create B  different datasets. A decision tree is then trained on each dataset before the predictions are aggregated by the empirical mean in Equation (1).

(1) $$\hat{f_{avg}}\left(x\right)=\frac{1}{B}\overset{B}{\underset{b=1}{\sum }}\hat{f_{b}}\left(x\right).$$

By using the empirical mean, the variance of the prediction is reduced. However, this variance reduction effect becomes smaller when each model is highly correlated. This is the case in bagging, where each of the decision trees will look quite similar. Random Forest [19] provides an improvement to bagging by decorrelating the decision trees. This is achieved by forcing the split to consider only a subset of the features of the data. While bagging increases accuracy over regular decision trees, it comes at the cost of interpretability. One can no longer directly inspect the model, as is the case for a single tree, since bagging aggregates results across models. However, it is still possible to obtain a summary of the importance of each feature.

To predict the class probabilities of an input sample in RF, one uses the mean of the class probabilities of the trees. The class probabilities of a single tree are the fraction of samples of the same class in a leaf.

#### 2.3.2. Model Selection

In this study, a binary classification problem was observed where one specific class was over-represented in the data. A stratified sampling was implemented to ensure the balance between the classes was preserved when splitting the data into train and validation sets. A cross-validation was then used to determine the set of hyperparameters of the model. The hyperparameters in question were as follows:

- The maximum depth of the decision trees;
- The numbers of features to consider when building a tree in the RF;
- The number of trees in the forest;
- The minimum number of samples required to split a node;
- The minimum number of samples to be considered as a leaf node in the tree.

The response from the model was in the form of binary variable, as specified in Table 1. Since the dataset was imbalanced with more negative (no failure) than positive (failure) samples, the results and metrics used for model selection should be carefully reported. In typical classification tasks, the *accuracy* of the predictions is defined using Equation (2).

(2) $$Accuracy=\frac{TP+TN}{TP+FP+TF+TN}$$

However, for an imbalanced dataset, the accuracy can be misleading. One could then obtain a high accuracy by only predicting the dominant class. To counteract this effect, the balanced accuracy was utilized (Equation (3)) as the metric, which is defined by the mean of the true positive rate (i.e., *Sensitivity*) and the true negative rate (i.e., *Specificity*).

(3) $$BalancedAccuracy=\frac{1}{2}\left(\frac{TP}{TP+FN}+\frac{TN}{TN+FP}\right)=\frac{Sensitivity+Specificity}{2}$$

#### 2.3.3. Pipe-Break Probability (P) Assessment

Pipe data and operational data for the network of Trondheim were obtained from the municipality. Historical pipe failure data were used to train the model to predict future failures. The following variables for the pipes were used as inputs to the RF model:

- Pipe dimensions, i.e., length and diameter and material;
- Maximum hydraulic pressure (data collected from hydraulic model);
- Number of buildings above or in close proximity to the pipe;
- Traffic above or in close proximity to the pipe;
- Age/installation year;
- Historical pipe failures/leakages.

**Figure 3:**
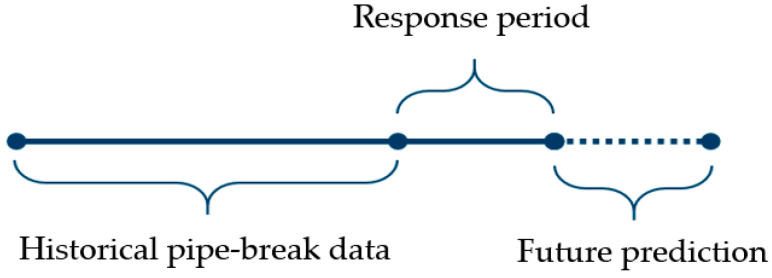
Schematic of pipe failure prediction by Random Forest.

Continuous data (where available) were used instead of grouping to facilitate better accuracy in the grouping of the pipes through the decision trees. Pipes that are no longer in operation and have been decommissioned were part of the analysis in RF to provide information on how pipes of different materials and ages behave with regard to pipe failures. For each pipe, the past five years were used as the response period. The number of failures before this five-year period was used as the historical failures measure in the input. These data were used together with the pipe parameters to predict the probability of pipe failure during the five-year horizon, as illustrated in Figure 3. When predicting on a new pipe, the probability of the pipe breaking within the next five years can be estimated. For training and testing of the model, the pipe dataset was split into training and validation sets with a 70/30 ratio. The model was selected based on the training data using cross validation to obtain the best hyperparameters. A stratified sampling, as explained in the previous section, was implemented to ensure a valid balance of the two classes. The validation dataset was used to test the model and establish the accuracy of the predictions.

### 2.4. Consequence Assessment (C)

#### 2.4.1. Hydraulic Model and AVAT Simulation

The hydraulic model of Trondheim WDN was based on the EPANET 2.2 demand-driven engine. Three separate models were developed to represent the three-service scenarios investigated in this study, i.e., service from J, B, and both (JB).

AVAT (Asset Vulnerability Assessment Tool) is a tool developed in the H2020 STOP-IT project (https://stop-it-project.eu/ accessed 17 December 2021). AVAT has the capability of assessing vulnerabilities of a WDN using several metrics, both probabilistic and deterministic, at the system and asset levels. Of particular interest in this study was the deterministic index at the asset level, namely the Link Critical Index (LCI), calculated in AVAT [31]. The LCI is a link/element index identifying the number of disconnected nodes due to an element outage in an undirected graph representation of the distribution system. The LCI of an asset (i.e., pipe, pump, or valve) is proportional to the number of disconnected nodes caused by its failure (Equation (4)). The LCI was used to assess the topological importance of each pipe in the WDN studied. AVAT requires a steady state EPANET simulation (i.e., ‘snapshot’ analysis) to perform its simulation. Given the deterministic nature of LCI calculation that is based on network topology, the outcome of AVAT simulation is not time-dependent, i.e., LCI value of a pipe is constant at any simulated time.

(4) LCI(i)=N({number of disconnected nodes if pipe i is disconnected})

#### 2.4.2. Calculation of Consequence—Link Hydraulic Criticality

Link Hydraulic Criticality (LHC) was introduced in this study as a measure of consequence that combines the ratio of disconnected nodes calculated by LCI over the total node number in the WDN and the flow conveyed by each pipe over the total flow of the WDN (Equation (5)). The flow data were taken from a demand-driven, extended-period hydraulic simulation (24-h) in EPANET 2.2 at 17.00 that corresponded to the peak of the diurnal pattern of water consumption in Trondheim. Given the mechanistic nature of LCI, a sensitivity test was conducted to assess the LHC value based on LCI, flow, and the combination of both, and at a different simulation time at 00.00 (see Appendix C).

(5) $$\mathrm{LHC}\left(i\right)=\frac{\mathrm{LCI}\left(i\right)}{total\;node}+\frac{\mathrm{flow}\left(i\right)}{total\;flow}$$

### 2.5. Risk Assessment (R)

#### 2.5.1. Risk Matrix Establishment

Risk matrix is a useful visualization method of displaying the interaction of probability and consequence to increase visibility of risk and to better assist decision making. Even though standard risk matrices exist in certain contexts, many a time, individual projects or organizations need to create their own tailor-made risk matrices, especially when determining the classes of the risk, to exercise prioritization of an action.

In this study, a risk matrix consisting of five levels of Probability (P) and six levels of Consequence (C), with increasing likelihood (P0–P4) and severity (C0–C5), respectively, was applied (Table 2). The numbers in the risk matrix cells represent the corresponding values of (P,C). The risk matrix was color-coded to reflect the risk levels as high (red), moderate (yellow), and low (green) by considering the multiplication product of P and C, described as the ‘PC value’ in Table 3.

The risk level classification applied in this study was subjectively determined with the goal of establishing initial groups of pipes to be prioritized in the rehabilitation plan. The classification of P and C, however, was conducted in a more analytical way. The values of P and C, calculated as described in Section 2.3.3 and Section 2.4.2, showed a specific distribution shape if sorted in decreasing order as a function of the number of pipes (see Appendix B). A linear classification was applied for the probability groups while a logarithmic classification was applied for the consequence groups, as defined in Table 4.

#### 2.5.2. Critical Pipe List for Rehabilitation

A list of pipes with the highest risk values, i.e., values of multiplication of P and C, was then established. Following this definition, it was possible to simply populate the three risk groups based on the PC values, i.e., values/color-coded risk groups, as outlined in Table 3.

## 3. Results

### 3.1. Probability Assessment of the Case Study

**Figure 4:**
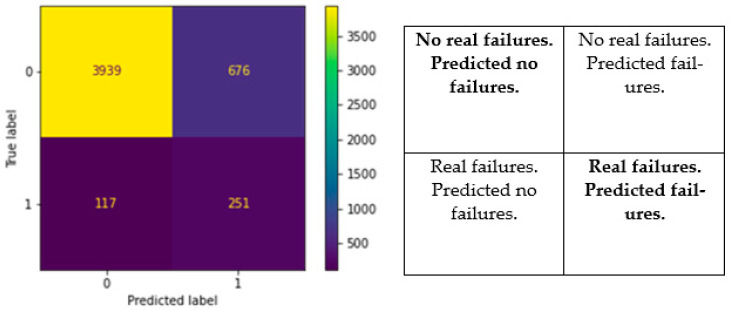
RF confusion matrix for the BA model.

Figure 4 shows the results from the model BA (Balanced Accuracy) that were tested using Random Forest analysis for the pipe data. The results are based on the testing of the BA model on a validation dataset, which consisted of 30% of the total number of pipes of the whole dataset.

The validation period, i.e., the length of the period on which the model was run in order to compare real failures with predicted failures, was 5 years. The meaning of the four quadrants follows the definition of actual vs. predicted interactions given in Table 1. The BA model predicted a total of 927 failures during the prediction period. This is the sum of the quadrants (predicted label = 1 and True label = 1) and (predicted label = 1 and True label = 0). The results further show the following:

- The model was able to predict 251 out of the total 368 actual failures happening in the period. This equals 68% of the actual failures.
- The model predicted no failures on 3939 pipes, when the real number of pipes with no failures was 4615 (3939 + 676). This is equal to 85.4% accuracy in the estimation of pipes not experiencing a failure.
- The model predicted that a higher number of pipes would experience a failure than what was recorded. The recorded data were biased, i.e., the real failures were dependent upon the observed failures being recorded.

**Figure A1:**
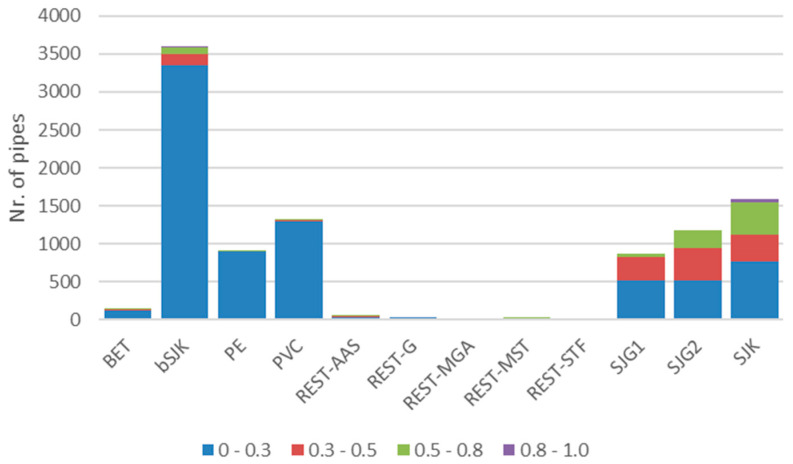
Number of pipes as a function of pipe diameter and material.

For the risk analysis, the BA model was used to represent the probability of failure on individual pipes. Appendix A presents detailed information on the pipe data supplied for the RF analysis. The importance of the pipe parameters for deciding the probability of pipe breaks is defined by the ‘Predictor importance’ factor in RF. The Predictor importance for the modelling of the pipes in Trondheim is illustrated in Figure A1 in Appendix A, where the x-axis is defined as ‘feature importance’. The feature importance for each parameter is a number between 0 and 1, and the sum of the feature importance for all parameters is 1 (or consequently 100%). It can be concluded that for the WDN investigated in this study, the five most important parameters for the prediction are:

1. Breakages in the past (27%);
2. Age of the pipe (18.5%);
3. Material of the pipe is unprotected ductile iron, often constructed before 1980 (14%);
4. Maximum hydraulic pressure during the day (9.5%);
5. Length of the pipe (8.5%).

The BA model was used to estimate pipe breakage probabilities for the next five years. Table 5 shows the accumulated results from this estimation, showing the average probability (between 0 and 1, where 1 = 100%) of breaks on different pipe materials. As seen in the table, the number of pipes (#9698) is fewer than that in the hydraulic model as indicated in Figure 1 (#10,669). This is simply because pipes shorter than 1 m were excluded from RF analysis. The results correspond well with the experience and knowledge the Norwegian water sector has from working with this topic for more than 20 years. The groups with the highest probability of failures are the groups of pipes most often connected with challenges and problems. The most challenging groups of pipes are unprotected ductile iron pipes (SJK) and grey cast iron pipes (SJG2) constructed in the post-World War 2 era. The second most problematic pipes are old grey cast iron pipes (SJG1) and pipes of varying materials (not extensively used, such as asbestos cement pipes or glass-fibre-reinforced plastic).

**Figure 5:**
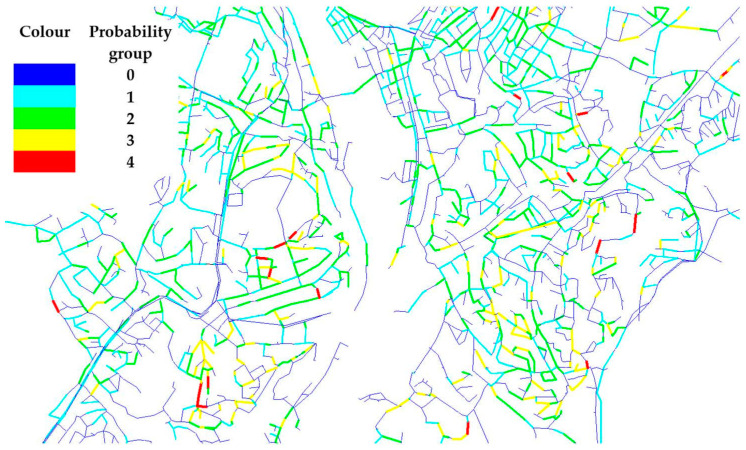
An excerpt of the WDN showing classification of pipes according to *p* values from RF-BA analysis.

*p* values were grouped into five different levels and the number of pipes corresponding to each group are shown in Table 6. The results were further used in the estimation of the risk of each pipe. The outcome of RF analysis shows that there are 47 pipes belonging to the group with highest *p* values during the next five years. The splitting of the probability in groups, as was the case in this study, shows that the number of pipes tends to increase as the probability decreases. To avoid sharing critical information, Figure 5 shows only an excerpt of the WDN with coloured pipes according to the probability classification to illustrate the locations of these pipes. The pipes with high *p* values are scattered across the WDN and the main supply pipes from the two sources do not fall into the category of pipes with high P. As seen also in the figure, pipes belonging to one segment do not necessarily exhibit same *p* values owing to the individual pipe data and RF simulation that can emphasize such differences. Certainly, there is just one set of *p* values from RF-BA analysis used in this study irrespective of the supply scenarios modelled, simply because the P is independent of supply scenarios and relies solely on pipe data.

### 3.2. Consequence Assessment of the Case Study

A sensitivity analyses for C valuation (i.e., LHC) was conducted. The test was conducted to assess contribution of different factors’ weights (LCI and flow) to the calculation of C. Table A2, Table A3 and Table A4 in Appendix C show the risk matrices produced from exercising the sensitivity test using supply scenario from “B” as an example. The risk matrices were compiled with C calculated with LCI only (C1), flow only (C2), and the combination of the two with an equal weight, i.e., 50% LCI and 50% flow in the pipe (C3). The classification of P was taken from RF-BA, as presented in the preceding section.

As seen from the tables, the sensitivity analysis returned different risk matrices for each C calculation method. For example, the pipes listed in the red list are different, as seen in Table A5. Risk calculated with C based on flow in the pipe only returned nine pipes, while C calculated by LCI and LCI-flow combination returned 12 pipes in the red list. This exercise shows a trade-off in how C value is evaluated. Given that LCI is a deterministic index, LCI overestimates the topological importance of a pipe, i.e., pipes with low flow may exhibit a higher importance compared to those with high flow. On the contrary, calculation of the consequence based on flow only may undermine the topological importance, e.g., a pipe with high flow but connected to fewer nodes may inflict higher importance compared to a pipe with lower flow supplying water to a larger number of nodes. Therefore, it is of paramount importance to combine the two factors (LCI and flow) in the assessment of C. This is certainly a more intuitive approach given that the risk-based rehabilitation method presented in this study aims to provide a more comprehensive image of hydraulic criticality of the pipes in the WDN (i.e., their topological and customer demand fulfilment importance) as a basis for prioritizing the rehabilitation plan.

**Figure 6:**
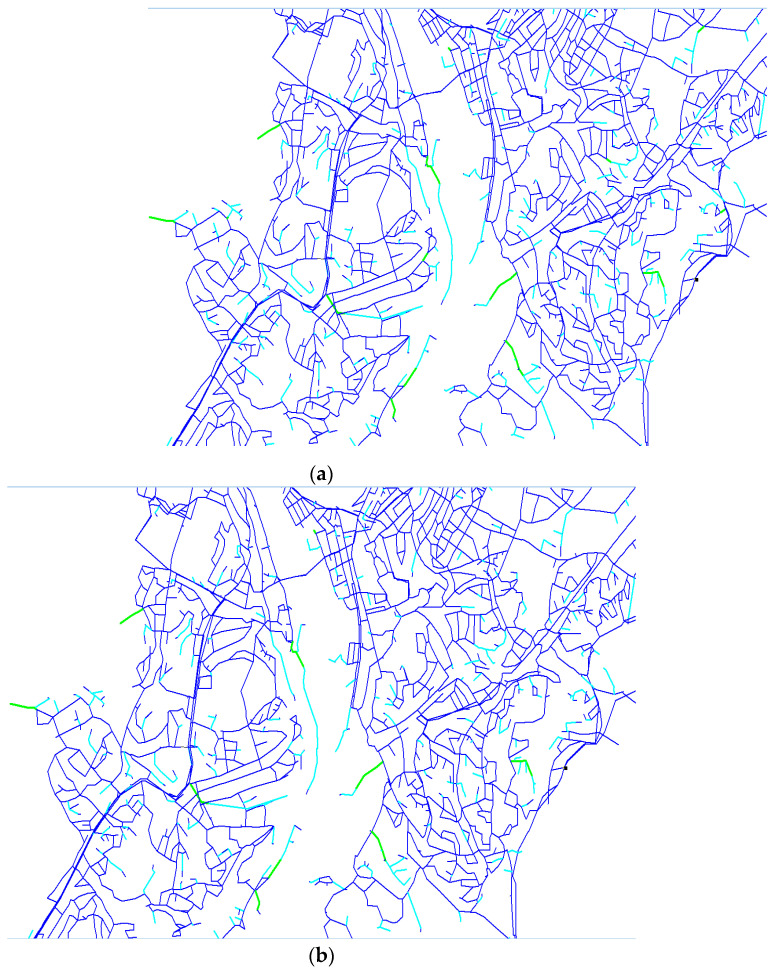

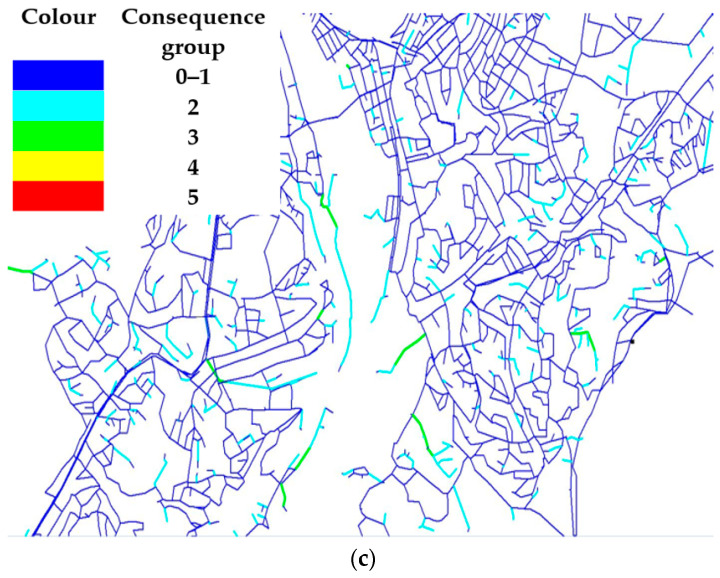
Classification of pipes for an excerpt of Trondheim WDN according to C values in different service scenarios with supply from (**a**) J, (**b**) B, and (**c**) JB.

Figure 6 shows the excerpts of Trondheim WDN and the results of AVAT simulations from the three service scenarios investigated in this study. The pipes are grouped based on their C values evaluated with equal weight of LCI and flow. At a glance, the figures do not seem to highlight any difference between the service scenarios tested at this level of magnification, and the consequence evaluation method applied in this study yields predominantly similar C scores for the service pipes (pipes other than the main pipes connecting the two sources to the WDN). However, there are, in fact, 107 pipes that are affected by the service scenarios detected by AVAT. This is indeed an interesting result from the C exercise showing a possible extension of the method by quantifying how much the LCI and/or flow are changing when different supply scenarios are imposed on the WDN.

Intuitively, the main pipes from the sources (not shown in the figures) indicate the highest C values owing to their topological and demand fulfilment importance, i.e., disconnection of these pipes results in higher numbers of disconnected nodes and unsupplied flows. The exercise also highlights the nature of exerting AVAT simulation. Trondheim WDN can be viewed as a hybrid system. Although the system is predominantly a loop/gridiron system, it exerts a degree of branch system characteristics in some parts of the WDN. It can be demonstrated that AVAT is able to detect such subtle transitions, but at the same time, exposes its tendency to assign higher topological importance to pipes connected in such a branch system. This is yet another argument underlining the importance of involving flow in pipes when conducting consequence assessment. It can be seen in the figure that some central pipes in the loop system do exhibit quite high C values.

### 3.3. Risk Assessment of the Case Study

Table 7a–c shows the risk classification of pipes obtained by combining the P and C values. As seen in the table, even though the different service scenarios return different numbers of pipes classified in the yellow and green risk groups, there are just a few pipes on the red list (13 pipes for the service scenario from “J” and 12 pipes for the service scenario from “B” and “JB”). Indeed, matching P and C values and arranging them in a matrix intuitively eases the interpretation of the risk values and how the two risk components interplay. Given that only one set of *p* values was adopted in the risk matrix for all service scenarios investigated in this study, it can be straightforwardly deduced that the differences in pipe numbers populated in each cell of the matrix are due to the C component. Indeed, as indicated in the table, the ‘sum row’ is constant and the ‘sum column’ is changing depending on the service scenario tested.

**Figure 7:**
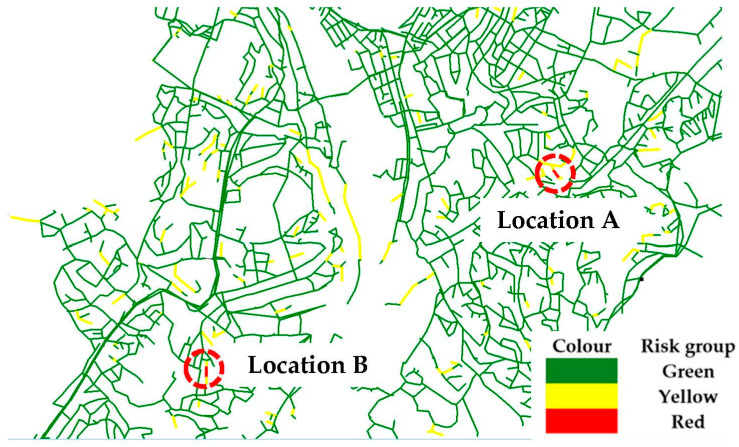
Locations of critical pipes from risk assessment for all service scenarios tested.

The three risk matrices can thus be crosschecked with the pipe data and visually shown in a figure that represents an important feature of the risk-based approach that is used to identify and help prioritize the pipe for the rehabilitation plan. For the sake of discussion in this section, the focus shall be put solely on the pipes in the red risk group for all service scenarios tested. A critical note on how the risk matrix can be better developed or interpreted is presented in the discussion section. Figure 7 shows an excerpt of Trondheim WDN with a couple of pipes in the ‘red’ group. Due to the sensitivity of the data, not all locations of the critical pipes can be shown in this paper, but it can be safely mentioned that the locations are spread throughout eight specific locations in the WDN.

As a general impression from the exercise, one can immediately observe a similar feature of risk plot in comparison to the pipe breakage probability plot in Figure 5, i.e., pipes belonging to a same segment/stretch do not necessarily incur equal risk levels, as opposed to plot of C values in Figure 6. Indeed, C valuation through AVAT and flow in pipes works on a hydraulic basis as opposed to how P is assessed, that is, based on pipe data. Hence, AVAT displays the tendency to give similar—or a gradation of—LHC for pipes in the same stretch or segment. While C is shown to be predominant in influencing the differences concerning pipe grouping in the risk matrix, P is predominant in dictating the location of such pipes. At a glance, this is not so trivial but, owing to the fact that P is based on pipe data, it is of course spatially bound. Hence, it is not at all surprising that all the critical pipes indicated in the risk matrix are found in locations where the *p* values are high. As mentioned above, the service scenario from “J” returns 13 red pipes as opposed to 12 pipes for the service scenario from “B” and “JB”. The difference in number is due to the different risk valuation of a pipe in one of the identified critical pipe locations in Trondheim WDN. The pipe is considered ‘red’ in scenario “J” but is valuated as a ‘yellow’ pipe in the other two service scenarios. This provides further evidence for this argument of spatially bound *p* values.

### 3.4. Pipe Rank for Rehabilitation

Table 8 identifies and ranks the pipes in the red risk group from each service scenario investigated in this study using a simple quantification of PC values as described in Section 2.5.2.

It should be straightforward to comprehend by now that the rank list returns similar pipe IDs at the same locations with different PC values owing to their differences in C values. Involving PC values in this exercise can seem too oversimplistic. Indeed, one can argue there are many factors that may weigh in and must be considered to finally arrive at the conclusion of pipe prioritization. The discussion section will provide such insights.

## 4. Discussion

Pipe failure statistics and probability assessment play a central role in a reliability analysis. It is, therefore, important to be able to test and verify the models that are used for probability assessment. In the study, the model was trained by 70% of the dataset verified with 30% of the data. This enabled the possibility to verify how accurately the model was able to predict failures on pipes that had real failures and predict no failures on pipes that had no real failures. The municipality experiences about 140 breaks per year. Over a period of the next five years (i.e., the estimation period in this study), this amounts to about 700 breaks, which means that the BA model predicts more (it predicted 927 failures) than the actual observed number of breaks. There is, therefore, a possibility that the BA model is over-estimating the number of failures. However, there is another more plausible explanation for the overestimation of failures. Even though the municipality have not registered a failure on a pipe, it does not mean that the pipe has a smaller or several smaller failures that the municipality is yet to discover. For a failure to be registered, it must be observed. Therein lies a bias in the numbers. Many of the pipes that were predicted to fail by Random Forest analysis, but for which failure was not observed reality (True label = 0 + Predicted label = 1), may have smaller leakages that are yet to be discovered, e.g., background leakages. Our interpretation of the results is that the BA model identified pipes with leakages that are yet to be discovered by the municipality. This means that there may be failures that are not yet observed. In the testing of the BA model, where the model was tested on the verification dataset, it showed an accuracy of 68% in terms of estimating failures on pipes that have actual failures in real life. This is important because it conveys something about the probability of estimating failures on pipes that will experience real failures in the next five years. The model was able to estimate 2 out of 3 failures that occurred on the validation dataset.

It is also important to note that the study found pipe failure history to be an important predictor, as was also found by other researchers [32,33], ahead of other predictors, e.g., age, material, length, and pressure [8,34,35,36]. Such a ‘clustering’ pipe failure phenomenon is well known and can be a result of, e.g., an inadequate repair of the previous failure [37,38].

In this study, the mechanical reliability analysis was represented by AVAT simulations that quantified the number of disconnected nodes as a function of pipe failure events. The application of AVAT in a large, real-life WDN comprising loop and branch pipes showed that AVAT has a tendency of giving away higher LCI scores for branch pipes and often undermines the centrality of pipes in complex pipe loops (see Figure 6). This highlights another common tendency for most of the reliability index methods besides, e.g., a greater criticality index of large pipes or pipes serving high-demand nodes [27]. Hence, one needs to include other parameter(s) to be able to improve the pipe criticality analysis. From the perspective of water utility providers, the objective is to supply water of adequate quantity and pressure. Hence, the number of unsupplied customers and/or pressure sufficiency can be an important factor. From AVAT simulation, one can obtain the number of disconnected nodes, which is quite straightforward for a branch system. For a loop system, the calculation of disconnected nodes will not be as straightforward as in a branch system. The unsupplied flow to a node due to disconnection of a pipe can be compensated by the node’s connection with other pipes in the WDN, i.e., by the rerouting of flow with a higher energy dissipation/headloss as compensation. To account for this issue, the study combined the hydraulic–topological reliability in a simple way through the inclusion of unsupplied flow if a particular pipe was out of service. This approach was shown generically to provide indication of the critical pipes; however, the unsupplied flow did not necessarily/directly reflect the true value of unsupplied demand for customers. One can argue that pressure-driven modelling may help in calculating flow and pressure with much greater flow accuracy but given that AVAT involves a steady-state hydraulic analysis, this may not amount to a huge difference. The difference, however, can be significant if one can evaluate the background leakage and decouple it from the real customer demand at nodes and, consequently, the leakage flow contribution to the total flow of water in a pipe, e.g., by means of using a well-calibrated hydraulic model coupled with a leakage algorithm [39,40] or by observing customer demand records [41]. A simpler solution may involve developing a separate disconnected nodes list and quantifying the customer demands attached to those nodes. Hence, the problem of the indirect representation of unsupplied demand by flow in pipes can be resolved.

As described in Section 2.4, the C assessment involved a hydraulic simulation at 17.00. To make sure the chosen time of simulation was representative, another sensitivity test was conducted using flow data at 00.00 and the result is presented in Table A6 (Appendix C). One can again observe that the ‘sum row’ is constant irrespective of the hydraulic simulation owing to the single set of *p* values used in the test. In addition, one may see that the ‘sum column’ is different if compared to that of 17.00. There is an increase in number of pipes in C0–C1 and a decrease in C2–C3, while the numbers in C4–C5 are constant. Indeed, some of the pipes in C2–C3 conveying lower flows at 00.00 compared to at 17.00 render lower C values and are demoted to the lower C classes. This does not, however, impact the number of pipes in the red risk group as the total pipe number belonging to the group is merely the same and still refers to the same critical pipe IDs as listed in Table A5.

The risk matrix approach applied in this study was found to help in improving the visualization of the risk events investigated. Nonetheless, the straightforward classification of risk levels in this study can be refined in many ways. Some of the criticisms of the application of risk matrices in decision making were addressed in this study. For example, the P and C assessment involves objective data that quantifies the two factors and, hence, minimizes cognitive/subjective biases that lead to off-target analysis [42]. A sensitivity analysis was also performed to assess how some specific model variables impact the output of the method applied. Only after an objective quantification or the risk levels can real life factors be considered deeply to better alleviate a false sense of security of the risk levels and ensure the effectiveness of risk treatment (e.g., setting up a pipe rehabilitation plan). For example, the water utility provider may perceive P and C differently; hence, the finalization of the risk matrix should be conducted in close interaction with the stakeholders. External data, e.g., data from other infrastructures that are directly or indirectly connected to the operational aspects of WDN, such as railways, wastewater pipes, and so on, can help assist better decision making. Some of these pieces of data were included in the RF analysis (e.g., the number of buildings and the traffic load above or near the pipe), but not, in a sense, evaluated when it came to the consequence analysis.

Traditionally, pipe rehabilitation plans in Norway are mostly based on pipe breakage probability assessment. This study took risk into consideration by combining the probability of a failure with the consequence if it happens. The main advantage with this approach is that pipes with high probability of failure are not necessarily prioritized if their failure does not involve a significant consequence. This allows the municipality to focus on high-risk pipes in the rehabilitation planning process.

## 5. Conclusions

A risk-based pipe rehabilitation planning method was developed based on a specific risk event, i.e., pipe breakage, in which the risk associated with the event was assessed using pipe data and a hydraulic model. The study demonstrated that the method is generic and applicable for a complex WDN. Moreover, the method should also be relevant for other cases. Risk assessment supports water utility providers, at the tactical decision level, in prioritizing pipes for rehabilitation work by ranking pipes in terms of risk severity associated with risk management objectives. A simplified approach was developed in this study for the risk assessment of WDNs according to the steps described in Section 1.

The approach was applied to a large water distribution system in Trondheim, Norway. From the conducted analysis, the following can be deduced:

- Random Forest analysis was able to provide a good prediction accuracy of pipe failure probability. By training the model using historical pipe data, the model was able to predict 68% of the actual failures and 85.4% of pipes not experiencing a failure in the verification period. The model predicted a higher number of pipes that would experience a failure than recorded/registered. Note that to be registered, a pipe failure must be observed. Hence, it is plausible that the model identified pipes with leakages that are yet to be discovered by the municipality, i.e., there may have been failures that are not yet observed.
- The Link Hydraulic Criticality (LHC) introduced in this study was a combined index calculated as an equal weighting of the ratio of the Link Criticality Index from AVAT and the total number of nodes in the WDN, and the flow conveyed by the corresponding pipes. LHC was able to evaluate the criticality of individual pipes under the different service scenarios presented.
- A risk matrix approach can be used to visualize the results from the Probability (i.e., Random Forest) and Consequence (i.e., LHC) parts and can provide the utility to better plan their rehabilitation process. By means of quantification, the elements of the risk matrix, as well as subjective biases in the risk assessment, can be avoided. Only after an objective quantification or the risk levels can real life factors be considered deeply to better alleviate a false sense of security of the risk levels and ensure the effectiveness of risk treatment (e.g., setting up a pipe rehabilitation plan).

Inclusion of other aspects, e.g., water quality and physical leakage, will prove necessary for the future studies to provide an even more comprehensive approach to help water utility providers set their pipe rehabilitation plan.

## Figures and Tables

**Table 1:** Adopted RF confusion matrix to assess the model accuracy.

	Predicted Negative	Predicted Positive
**Actual negative**	True Negative (TN)	False Positive (FP)
**Actual positive**	False Negative (FN)	True Positive (TP)

**Table 2:** Risk matrix showing classification of pipes based on their combined (P,C) values.

		Consequence (C)					
		C0	C1	C2	C3	C4	C5
Probability (P)	P4	(4,0)	(4,1)	(4,2)	(4,3)	(4,4)	(4,5)
	P3	(3,0)	(3,1)	(3,2)	(3,3)	(3,4)	(3,5)
	P2	(2,0)	(2,1)	(2,2)	(2,3)	(2,4)	(2,5)
	P1	(1,0)	(1,1)	(1,2)	(1,3)	(1,4)	(1,5)
	P0	(0,0)	(0,1)	(0,2)	(0,3)	(0,4)	(0,5)

**Table 3:** Color coding in risk matrix.

Risk Group	PC Value
Red	8–20
Yellow	2–6
Green	0–1

**Table 4:** Classification of P and C values applied in the study.

Probability Group	Probability Value	Consequence Group	Consequence Value
P0	P < 0.20	C0	C < 1.10^−5^
P1	0.20 ≤ P < 0.40	C1	1.10^−5^ ≤ C < 1.10^−4^
P2	0.40 ≤ P < 0.60	C2	1.10^−4^ ≤ C < 1.10^−3^
P3	0.60 ≤ P < 0.80	C3	1.10^−3^ ≤ C < 1.10^−2^
P4	P ≥ 0.80	C4	1.10^−2^ ≤ C < 1.10^−1^
		C5	C ≥ 1.10^−1^

**Table 5:** Predicted failure probability in 5 years based on pipe material groups.

Group	Number of Pipes	Length (m)	Average P (%)	Predicted Failure Probability per Pipe Length (km^−1^)
SJG1	875	63,144	27.87	3.9
REST	121	34,229	19.51	0.7
SJK	1588	122,484	36.79	4.8
SJG2	1185	93,481	35.59	4.5
bSJK	3582	289,476	10.76	1.3
PVC	1308	144,327	4.97	0.5
BET	137	29,359	18.44	0.9
PE	902	84,774	3.32	0.4
Total	9698	861,273	18.34	2.1

**Table 6:** Groups of pipes based on *p* values from RF-BA analysis.

Probability Group	Probability	Number of Pipes
0	P < 0.20	6261
1	0.20 ≤ P < 0.40	2089
2	0.40 ≤ P < 0.60	950
3	0.60 ≤ P < 0.80	351
4	P ≥ 0.80	47
	Sum	9698

**Table 7:** Risk matrices of pipes based on their combined failure probability and consequences for service scenarios (**a**) J, (**b**) B, and (**c**) both J and B.

(a)								
		**Consequence (C)**						
		**0**	**1**	**2**	**3**	**4**	**5**	**Sum Row**
Probability (P)	4	40	0	4	3	0	0	47
	3	311	12	25	1	2	0	351
	2	832	34	71	10	3	0	950
	1	1780	82	185	27	15	0	2089
	0	5195	266	626	132	40	2	6261
sum column		8158	394	911	173	60	2	9698
**(b)**								
		**Consequence (C)**						
		**0**	**1**	**2**	**3**	**4**	**5**	**Sum Row**
Probability (P)	4	40	0	4	3	0	0	47
	3	311	14	23	2	1	0	351
	2	832	40	69	7	2	0	950
	1	1780	90	180	25	14	0	2089
	0	5194	291	610	118	23	25	6261
sum column		8157	435	886	155	40	25	9698
**(c)**								
		**Consequence (C)**						
		**0**	**1**	**2**	**3**	**4**	**5**	**Sum Row**
Probability (P)	4	40	0	4	3	0	0	47
	3	311	12	25	2	1	0	351
	2	832	36	70	10	2	0	950
	1	1780	87	182	26	14	0	2089
	0	5193	280	617	124	21	26	6261
sum column		8156	415	898	165	38	26	9698

**Table 8:** List of pipes in the red risk group from each service scenario and ranking for rehabilitation based on the PC value.

Rank	Pipe ID	Service from “J”	Service from “B”	Service from “JB”	Pipe Length (m)	Diameter (mm)
1	T6844	12	12	12	279.7	150
2	T2756	12	12	12	200.3	150
3	T7650	12	12	12	281.1	150
4	T2255	12	12	12	275.7	250
5	T2813	12	9	9	47.4	250
6	T6577	9	9	9	69.4	150
7	T1160	8	8	8	100.5	150
8	T3674	8	8	8	111.6	150
9	T5570	8	8	8	158.3	200
10	T459	8	8	8	196.1	150
11	T2242	8	8	8	186.1	200
12	T2243	8	8	8	81.3	200
13	T3782	8	-	-	239.1	250

**Figure A2:**
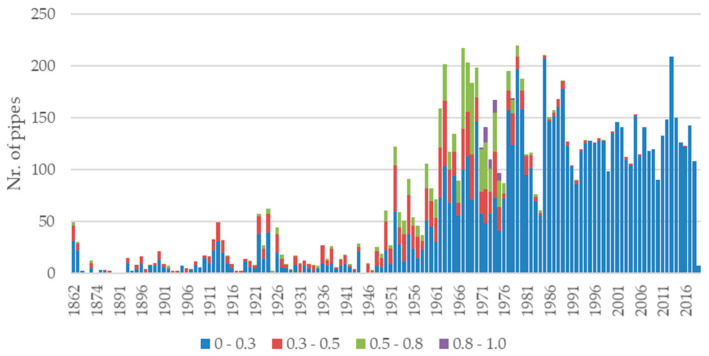
Number of pipes as a function of pipe diameter and installation year.

**Figure A3:**
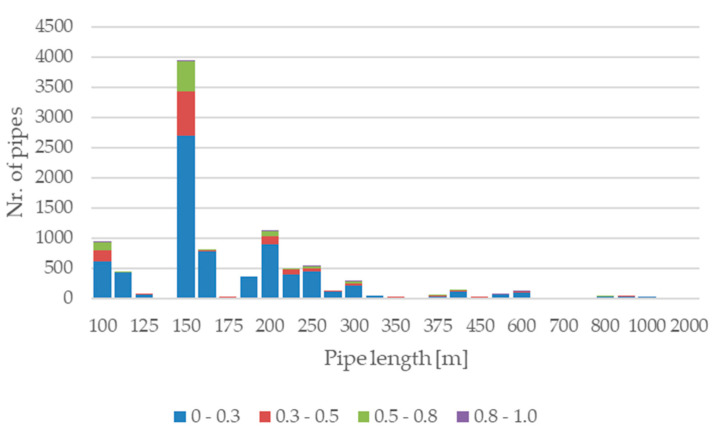
Number of pipes as a function of pipe diameter and pipe length.

**Figure A4:**
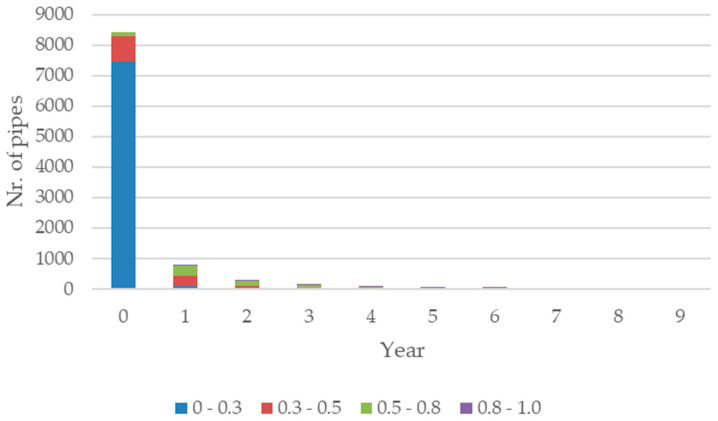
Number of pipes as a function of pipe diameter and earlier recorded failure event.

**Figure A5:**
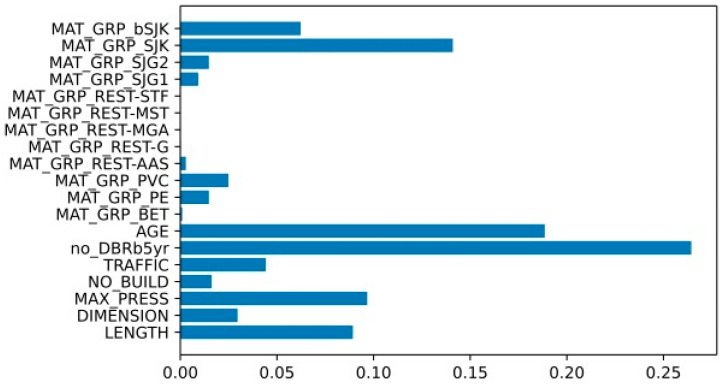
Schematic of pipe failure prediction by Random Forest.

**Figure A6:**
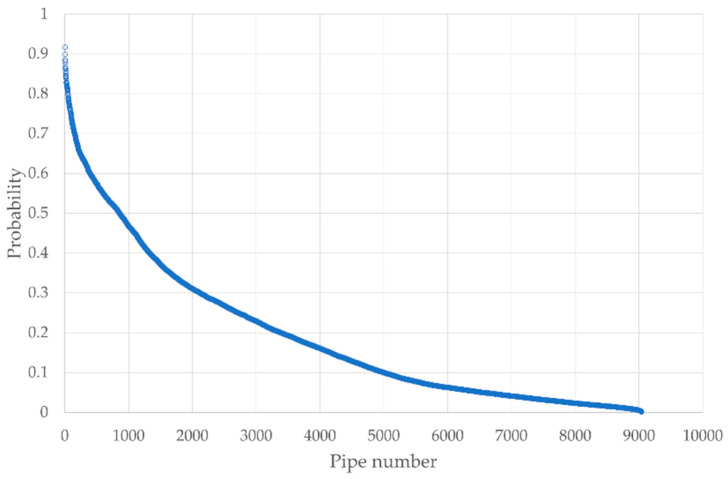
Plot of probability values from RF analysis sorted from highest to lowest values.

**Figure A7:**
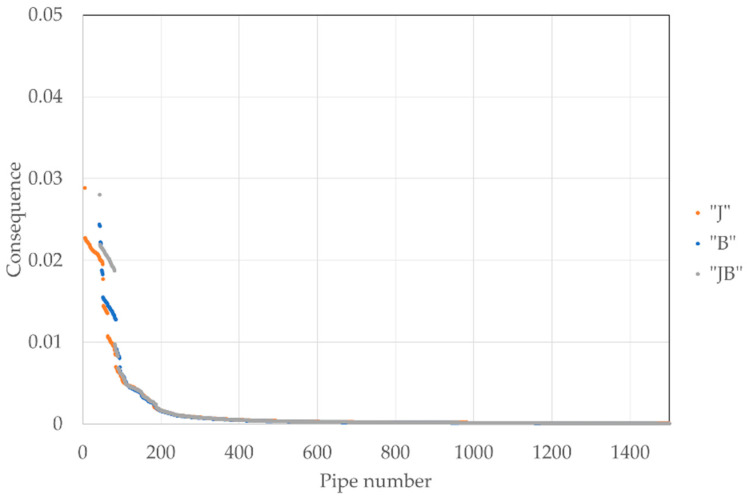
Plot of consequence values sorted from highest to lowest values.

## Data Availability

Not applicable.

## Appendix A

Pipe data for probability assessment by Random Forest.

**Table A1:** Grouping of pipes based on pipe metadata (material, age/installation year).

Material Group	Material Code	Comments
BET	BET	
bSJK	SJK	SJK with protection; installation year 1976–2019
PE	PE *	All PE code
PVC	PVC	
REST-AAS	AAS	
REST-G	GRP, GUP	
REST-MGA	MGA	
REST-MST	MST	
REST-STF	STF	
SJG1	SJG	Installation year 1862–1944
SJG2	SJG	Installation year 1945–1974
SJK	SJK	SJK without protection; installation year 1943–1975

* Represents All PE code.

## Appendix B

Sorted plots of Probability (from RF analysis) and Consequence (LHC from AVAT and hydraulic simulation) values for P and C classification.

## Appendix C

Risk matrices and critical pipe list produced from the sensitivity test for Consequence (LHC) evaluation using supply from “B” as an example.

**Table A2:** Risk matrix based on LCI only at time 17.00.

		Consequence (C)						
		0	1	2	3	4	5	Sum Row
Probability (P)	4	40	0	7	0	0	0	47
	3	311	0	37	2	1	0	351
	2	832	0	107	9	2	0	950
	1	1780	0	262	33	14	0	2089
	0	5194	0	900	118	25	24	6261
sum column		8157	0	1313	162	42	24	9698

**Table A3:** Risk matrix based on flow only at time 17.00.

		Consequence (C)						
		0	1	2	3	4	5	Sum Row
Probability (P)	4	40	1	3	3	0	0	47
	3	312	19	18	1	1	0	351
	2	837	52	57	3	1	0	950
	1	1800	129	130	27	3	0	2089
	0	5261	391	465	112	6	26	6261
sum column		8250	592	673	146	11	26	9698

**Table A4:** Risk matrix based on LCI and flow (equal weight, 50%) at time 17.00.

		Consequence (C)						
		0	1	2	3	4	5	Sum Row
Probability (P)	4	40	0	4	3	0	0	47
	3	311	14	23	2	1	0	351
	2	832	40	69	7	2	0	950
	1	1780	90	180	25	14	0	2089
	0	5194	291	610	118	23	25	6261
sum column		8157	435	886	155	40	25	9698

**Table A5:** Pipes on the red list in the service scenario from B calculated with different consequence variables.

LCI Only	Flow Only	LCI and Flow
T6844	T6844	T6844
T2756	-	T2756
-	-	T3674
T1160	-	T1160
T3674	T3674	-
T459	T459	T459
T7650	T7650	T7650
T5570	T5570	T5570
T2813	T2813	T2813
T6577	-	T6577
T2255	T2255	T2255
T2242	-	T2242
T2243	-	T2243
-	T2756	-
-	T3782	-

**Table A6:** Risk matrix based on LCI and flow at time 00.00.

		Consequence (C)						
		0	1	2	3	4	5	Sum Row
Probability (P)	4	40	0	4	3	0	0	47
	3	311	15	22	2	1	0	351
	2	832	51	59	6	2	0	950
	1	1781	106	168	20	14	0	2089
	0	5204	347	556	106	23	25	6261
sum column		8168	519	809	137	40	25	9698
